# Supplementary material for: HIVprotI: an integrated web based platform for prediction and design of HIV proteins inhibitors
Source: J Cheminform. 2018 Mar 9;10:12. doi: 10.1186/s13321-018-0266-y (PMC5845081; doi:10.1186/s13321-018-0266-y)
Supplement: Supplementary file 2 — Additional file 2. Source code of HIVProtI web server. [file 13321_2018_266_MOESM2_ESM.zip › HIVprotI_Source-code/hivprotI/weka-3-6-6/doc/weka/gui/treevisualizer/PlaceNode2.html]

PlaceNode2


---


|  |  |  |
| --- | --- | --- |
| **Overview** \| **Package** \| **Class** \| **Tree** \| **Deprecated** \| **Index** \| **Help** \| **Weka's home** | |  |
| **PREV CLASS**   **NEXT CLASS** | **FRAMES**    **NO FRAMES**     **All Classes** |  |
| SUMMARY: NESTED \| FIELD \| CONSTR \| METHOD | DETAIL: FIELD \| CONSTR \| METHOD |  |


---


## weka.gui.treevisualizer Class PlaceNode2

```
java.lang.Object
  weka.gui.treevisualizer.PlaceNode2
```

**All Implemented Interfaces:**: NodePlace

---

``` public class PlaceNode2 extends java.lang.Object implements NodePlace ```

This class will place the Nodes of a tree.

It will place these nodes so that they fall at evenly below their parent.
It will then go through and look for places where nodes fall on the wrong
side of other nodes
when it finds one it will trace back up the tree to find the first common
sibling group these two nodes have
And it will adjust the spacing between these two siblings so that the two
nodes no longer overlap.
This is nasty to calculate with , and takes a while with the current
algorithm I am using to do this.

**Version:**
:   $Revision: 1.4 $

**Author:**
:   Malcolm Ware (mfw4@cs.waikato.ac.nz)

---

**Constructor Summary**

`PlaceNode2()`


| **Method Summary** | |
| --- | --- |
| `void` | `place(Node r)`             The Funtion to call to have the nodes arranged. |

**Methods inherited from class java.lang.Object**

`equals, getClass, hashCode, notify, notifyAll, toString, wait, wait, wait`

**Constructor Detail**

### PlaceNode2

```
public PlaceNode2()
```


**Method Detail**

### place

```
public void place(Node r)
```

:   The Funtion to call to have the nodes arranged.

    :   **Specified by:**: `place` in interface `NodePlace`
    :   **Parameters:**: `r` - The top node of the tree to arrange.


---


|  |  |  |
| --- | --- | --- |
| **Overview** \| **Package** \| **Class** \| **Tree** \| **Deprecated** \| **Index** \| **Help** \| **Weka's home** | |  |
| **PREV CLASS**   **NEXT CLASS** | **FRAMES**    **NO FRAMES**     **All Classes** |  |
| SUMMARY: NESTED \| FIELD \| CONSTR \| METHOD | DETAIL: FIELD \| CONSTR \| METHOD |  |


---
